# Probing the Impact of Prematurity on Segmentation Abilities in the Context of Bilingualism

**DOI:** 10.3390/brainsci13040568

**Published:** 2023-03-28

**Authors:** Elena Berdasco-Muñoz, Valérie Biran, Thierry Nazzi

**Affiliations:** 1 Integrative Neuroscience and Cognition Center, CNRS & Université Paris Cité, 75006 Paris, France; thierry.nazzi@parisdescartes.fr; 2 Neurodevelopmental and Neurovascular Disorders Unit, Inserm & Université Paris Cité, 75019 Paris, France; valerie.biran@aphp.fr

**Keywords:** preterm infants, bilingualism, word segmentation, French

## Abstract

Infants born prematurely are at a high risk of developing linguistic deficits. In the current study, we compare how full-term and healthy preterm infants without neuro-sensorial impairments segment words from fluent speech, an ability crucial for lexical acquisition. While early word segmentation abilities have been found in monolingual infants, we test here whether it is also the case for French-dominant bilingual infants with varying non-dominant languages. These bilingual infants were tested on their ability to segment monosyllabic French words from French sentences at 6 months of (postnatal) age, an age at which both full-term and preterm monolinguals are able to segment these words. Our results establish the existence of segmentation skills in these infants, with no significant difference in performance between the two maturation groups. Correlation analyses failed to find effects of gestational age in the preterm group, as well as effects of the language dominance within the bilingual groups. These findings indicate that monosyllabic word segmentation, which has been found to emerge by 4 months in monolingual French-learning infants, is a robust ability acquired at an early age even in the context of bilingualism and prematurity. Future studies should further probe segmentation abilities in more extreme conditions, such as in bilinguals tested in their non-dominant language, in preterm infants with medical issues, or testing the segmentation of more complex word structures.

## 1. Introduction

Preterm birth is a serious public health issue which represents 10% of births and is a leading cause of neonatal death worldwide [1]. Given that full-term gestation corresponds to 40 gestational weeks (GWs), infants are considered preterm if they are born before 37 GWs. Preterm infants can be subdivided into extreme (<28 GWs), very (28 to <32 GWs), moderate (32 to <34 GWs), and late (34 to <37 GWs) preterms. In recent decades, significant advances in perinatal and neonatal care have improved the survival rates of preterm infants, leading to increased interest in the cognitive developmental outcomes of this population. Studies on preterm children have often found reduced cognitive abilities and higher risks for developmental delay than in full-term populations, e.g., [2,3,4,5], although no delay was found in [6]. These difficulties include language deficits, e.g., [7,8,9,10,11] (but see [12] for a study failing to find deficits), related to different linguistic domains, such as the phonological [13], lexical [5,13,14,15,16,17,18,19,20], and syntactic [13,21,22] domains. Importantly, links have been found between gestational age and birth-weight on the one hand and preterms’ language performance on the other hand, e.g., [4,8,11,16,19,21]. In order to better understand the language difficulties that preterms may develop later in life, it is crucial to identify language deficits in these infants as early as possible. Accordingly, the present study compared preterm and full-term 6-month-old infants’ ability to segment words from fluent speech, an important prerequisite to word learning. We tested infants being raised in bilingual environments, a situation that might incite further challenges on language acquisition for preterm infants.

In the following sections, we first provide an overview of research on language development in monolingual preterm infants during the first year of life. We then reflect on how bilingualism might impact early language acquisition in preterms. Studies on preterm infants compare them to full-term infants of the same postnatal age (calculated from the actual day of birth) to evaluate the role of language exposure and to younger full-term infants of the same maturational age (calculated according to due date of birth) to evaluate the role of prematurity.

### 1.1. Early Phonological Acquisition in Monolingual Preterms

Studies on early prosodic/suprasegmental acquisition have repeatedly reported deficits in preterm infants. First, while full-term infants discriminate between two languages with similar rhythms by 4 months in behavioral studies [23,24] and by 6 months in electrophysiological studies [25], such ability was found in preterm infants at 6 months maturational age [25,26] but not at 6 months postnatal age [25]. Similarly, while full-term infants are able to discriminate between different word stress patterns at 4 months [27] and prefer the predominant stress pattern of their native language by 6 months [28], preterm infants failed in both tasks when tested at the matching maturational age [27]. Finally, newborns’ ability to discriminate between forward and backward speech, taken as evidence of prosodic processing, was found to be linked to gestational age at birth in a group of infants born between 23 to 41 weeks of gestation [29]. These results point to an early disadvantage in prosodic processing in preterm infants.

Regarding phonetic/segmental processing (consonants and vowels), mixed findings have been found. Studies on vowels showed that preterm infants at 4 months maturational age fail to discriminate a vocalic contrast present in their native language (/dodi/-/dudi/), which full-term 4-month-olds are able to discriminate [30]. Moreover, decline in sensitivity to a non-native vowel contrast was not found at 12 months postnatal age in very preterm infants, contrary to 12-month-old full-term infants [31]. Studies on consonants show that preterm infants at 6 months postnatal age detect a consonant contrast present in their native language (/banan/-/panan/), similarly to full-term 6-month-olds [32]. As for a decline in sensitivity to non-native contrasts, a delay was found for very preterm infants tested with ERPs on a Hindi voiced dental-retroflex contrast (/da/-/Da/) [33], while no delay was found for very preterm infants tested behaviorally on an equivalent Hindi voiceless dental-retroflex contrast (/ta/-/Ta/) [34]. These findings suggest a delay in acquisition of segmental phonology for preterm infants, which might be more marked for vowels than for consonants.

Regarding phonotactics (that is, regularities in segmental combinations for words of a given language), perceptual experiments have shown evidence that both preterm and full-term infants begin acquiring phonotactic knowledge at 10 months postnatal age if they grow up in high-SES families [35] and at 12 months postnatal age if they grow up in low-SES families [34].

The above findings suggest that monolingual preterms have some difficulties regarding phonological acquisition. These difficulties seem to be more marked for prosody and vowels than for consonants and may be related to brain immaturity [25], a shortened prosodic exposure in utero [27], asynchronies in development [35], and/or atypical pathways of language acquisition [20]. Would such difficulties extend to other levels of language acquisition?

### 1.2. First Findings on Segmentation Abilities in Monolingual Preterms

One domain that has started to be explored is the ability to retrieve word forms from fluent speech. Investigating these word segmentation abilities is important because they lie at the intersection of phonological and lexical acquisition. Word segmentation is a requirement for lexical acquisition, since speech is a fluent stream with few silences between words and a range of cues (such as rhythmic cues, allophonic variations, coarticulation, phonotactic regularities, and transitional probabilities) each partly marking word boundaries. Previous research has established that segmentation abilities emerge around 6 months in several languages, mostly using a classical behavioral task, the Headturn Preference Procedure (HPP), that will also be used in the current study, or less often event-related potentials (for English, [36,37]; for French, [38]). It was also established that better segmentation abilities in infancy are related to larger vocabularies later in development [39,40,41,42,43].

A first study on Spanish- or Catalan-learning infants compared full-term and preterm infants on their ability to segment complex monosyllabic words at 8 months postnatal age [26]. Results showed that full-term infants could segment but failed to find evidence in preterm infants. Failure might have been due to difficulty of the task, in terms of duration of familiarization phase (45 s), too many test trials (16), or complex word structures (CVC/CCVC). Alternatively, it should be noted that full terms in that study showed a novelty effect, whereas a familiarity effect is usually found in this paradigm. This novelty effect could be interpreted as evidence that the full terms found the task easy. If the task was less easy for the preterms, they might have been moving from a familiarity to a novelty preference, resulting in a group level null effect. A second study reassessed segmentation abilities in preterm infants, testing healthy French-learning extremely-to-late preterm infants at 6 months of postnatal age, using a shorter familiarization phase (30 s), fewer test trials (12), and simpler word structures (CV). The authors found a significant segmentation effect marked by familiarity [44], as was found for full-term infants at the same postnatal [38] and maturational [44] ages, establishing syllabic segmentation as a core segmentation procedure in French-learning infants. Both studies on preterm infants also explored whether segmentation performance was related to neonatal variables such as gestational age or birth weight, but no significant links were reported.

### 1.3. The Present Study: New Segmentation Data and Extension to Bilingual Preterms

The present study will follow up on [44] and test whether monosyllabic CV word segmentation found in that study further extends to bilingual infants. Recent studies on early simultaneous bilingualism show that learning two languages affects the early developmental trajectory of language acquisition but does not lead to major delay or difficulties (for a review, see [45]). However, many parents still have concerns about raising their infant bilingually, as this means that their infant needs to learn two linguistic systems in parallel while receiving less input for each language overall, given that their linguistic input is shared between the two languages to be acquired. These concerns are augmented when an infant is born preterm and is thus at risk of cognitive and language development difficulties. Therefore, it is important to explore the potential interaction between preterm birth and bilingualism. To the best of our knowledge, no study has investigated the possible influence of bilingualism on early language acquisition in preterm infants. Yet, a few studies have explored the impact of bilingualism on segmentation abilities in full-term infants. Those studies have shown that bilingual full-term infants show segmentation abilities at similar ages as their monolingual peers (for English/Mandarin 7- to 11-month-olds, [46]; for Spanish/Catalan 6- and 8-month-olds, [47]; for French/English learning 8-month-olds, [48,49]). One study found even earlier segmentation in the case of specific word structures present in their second language (for English–Spanish 8-month-olds, [50]). Lastly, across these prior studies, segmentation effects have always been found in the dominant language, and, when tested, no clear dominance effects were found.

The present study explores segmentation abilities in (1) healthy bilingual extremely-to-late preterm infants at 6 months postnatal age and (2) full-term bilingual 6-month-olds, in order to foster further knowledge about possible interactions of bilingualism and prematurity in the emergence of word segmentation abilities. Investigating these word segmentation abilities is important because they lie at the intersection of phonological and lexical acquisition. All infants tested in the present study were born hearing between 50 and 75% French with varying second languages, reflecting the diversity of the Paris area. Infants were tested with the HPP. They were familiarized with two passages, each containing a target word. They were then presented with repetitions of the two target words in isolation, and repetitions of two control words not heard in familiarization. In such a design, segmentation is attested by longer orientation times to the target versus the control words. Following the above-mentioned studies showing word segmentation abilities in bilinguals, we predict successful segmentation for the bilinguals born full-term. This is further supported by fact that the task we are using appears to be easy for monolingual infants of the same age [38]. Regarding the preterm bilinguals, the outcome will depend on whether premature birth has a negative impact on acquiring segmentation abilities in two languages rather than one, given that monolingual preterms are able to perform word segmentation.

## 2. Method

### 2.1. Participants

The full-term participants were 24 infants (13 girls and 11 boys), with a mean age of 6 months and 15 days (range: 6 months and 1 day to 6 months and 29 days). The preterm participants were 19 infants (9 girls and 10 boys), with a mean postnatal age of 6 months and 16 days (range: 6 months to 6 months and 26 days) or a mean maturational age of 4 months and 8 days. Of the 19 infants, 13 were extremely/very preterm infants (born < 32 GWs), and 6 were moderate/late preterm infants (born ≥ 32 GWs; see Table 1). Sample sizes are equivalent to previous studies on this issue, including the two similar studies having tested preterm and full-term monolingual infants [38,39,40,41,42,43,44].

All infants were recruited through the birth lists issued by the Paris city registry office to the last author or via a hospital (Hôpital Robert Debré, Paris). All parents gave informed consent before participation. Infants were included if they were exposed to French and another language and if their parent-estimated rate of exposure to French was between 50–75 percent on a daily basis (for participants’ other-language exposure, see Table 2). Only infants without neuro-sensorial impairments (hearing loss, retinopathy of prematurity, or cerebral lesions) were included. Infants’ linguistic and medical backgrounds were assessed using an information sheet, an interview, and the medical information provided in the child’s health record.

Nine additional preterm infants did not complete the experiment due to failure to consistently turn their heads (*n* = 3), fussiness or distraction (*n* = 1), crying (*n* = 3), parental interference (*n* = 1), or a segmentation index more than 2 *SD* above or below the group mean (*n* = 1). To examine whether excluded preterm infants differed from included preterm infants in gestational age and birth-weight, *t*-tests were performed (see Table 1). No significant differences were found between the two groups.

### 2.2. Stimuli

We used the same stimuli as in Experiment 1 of [38] and in the two experiments of [44]. Target words were 4 nouns with a CV syllabic structure and, as done in previous research on this topic, relatively low frequencies: /di/ = 4.86 (dit [a saying]), /po/ = 32.3 (pot [pot]), /te/ = 44.19 (thé [tea]), /gu/ = 124.8 (goût [taste]; frequency values are given per 1 million occurrences and calculated over a database of 31 million occurrences in the adult database LEXIQUE 2 [51]). Moreover, these words were not listed in the French MCDI (MacArthur Communicative Development Inventory, [52]). For each target word, an 8-sentence passage was created for the familiarization phase. The target words appeared either toward the beginning (4 times) or toward the end (4 times) of the sentences, and the mean number of syllables per sentence was 10.

The passages were produced by a female native speaker of French in a sound-attenuated booth. She first recorded the 4 passages in mild infant-directed speech for the familiarization phase and then 20 repetitions of each word for the test phase. The 4 passages and 4 lists of repeated words all lasted 20 s each.

### 2.3. Procedure, Apparatus and Design

The procedure, apparatus, and design were the same as in Experiment 1 of [38] and in the two experiments of [44]. The experiment was conducted in a sound-attenuated booth, which contained a 3-sided test booth made of pegboard panels. The test booth had a red light and loudspeakers (Sony xs-F1722) mounted on each of the side panels and a green light mounted on the central panel. A video camera was situated directly below the center light to monitor infants’ behavior. However, due to ethical reasons, videos were not saved, and coding was performed online. A PC computer terminal (Dell OptiPlex), audio amplifier (Marantz PM4000), TV screen, and response box were located outside the sound-attenuated room.

Each infant was held on a caregiver’s lap, and the caregiver was seated in a chair at the center of the test booth. Each trial began with the green light on the center panel blinking until the infant had oriented in that direction. The center light was then extinguished, and the red light above the loudspeaker on one of the side panels began to flash. When the infant had made a headturn bringing their head direction within 30° from the light/loudspeaker, the stimulus for that trial was played; the red light continuing to flash for the entire duration of the trial. Each stimulus was played to completion or stopped immediately after the infant failed to maintain the 30° headturn for 2 s. If the infant turned away from the red light for less than 2 s and then turned back again, the trial continued but the time spent looking away was not included in the orientation time (OT). Thus, the maximum OT for a given trial was the duration of the entire speech sample (20 s). If the infant’s initial OT was shorter than 1.5 s on a given trial, the trial was immediately replayed from the beginning, and the initial OT was discarded.

Each experimental session began with a familiarization phase in which infants heard 2 passages on alternating trials until they accumulated 30 s of OTs for each one. When the infants reached the familiarization criterion for one passage, the second passage continued to be presented until its criterion was also reached. The side of the loudspeaker from which the stimuli were presented was varied randomly from trial to trial. The test phase began immediately after the familiarization criterion was reached. This test phase consisted of 3 test blocks, in each of which the 4 lists of isolated monosyllabic words were presented. The order of the lists within each block was randomized.

Each infant was familiarized with 2 passages and tested with 2 target and 2 control monosyllabic words. The two words that were used as targets was counterbalanced for both infant subgroups (preterms and full-terms). Hence, in this design, any overall preference for target words cannot be attributed to intrinsic properties of the words (e.g., their frequency in the language or their acoustic properties) but can only result from their status with respect to familiarization (targets presented during familiarization, versus controls not presented during familiarization).

## 3. Results

**Figure 1:**
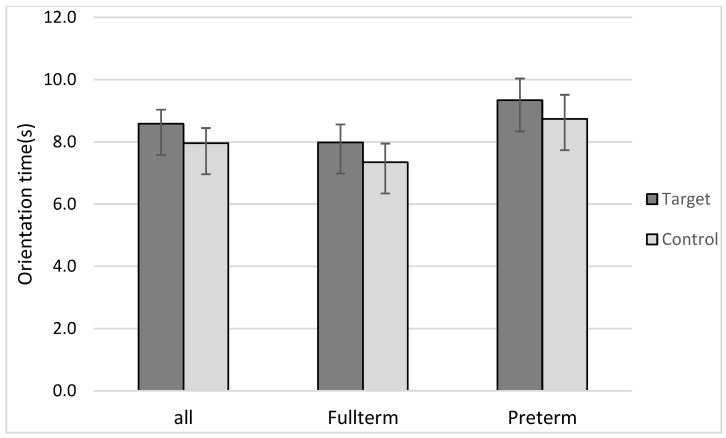
Mean orientation times (OTs) for all bilingual infants (**left**), full-term infants (**middle**) and preterm (**right**) for target and control words.

All analyses were conducted using SPSS 25 and were similar to those conducted in [38,44]. Mean orientation times (OTs) were calculated for the lists containing the target versus control monosyllabic words (see Figure 1). Given that the data did not differ from a normal distribution, as established by Shapiro–Wilk tests (all *p*s > 0.05), a repeated-measures ANOVA with mean OTs as the dependent measure, group (full-term versus preterm) as a between-group factor, and familiarity (target versus control) as a within-group factor was conducted. The effect of familiarity was significant (*F*_(1,41)_ = 5.74; *p* = 0.021, $\eta_{p}^{2}$ = 0.123), infants having longer OTs to target (*M* = 8.58 s, *SD* = 2.94) than to control (*M* = 7.95 s, *SD* = 3.18) words, indicating successful word segmentation. This pattern of preference for target words was found in 30 of the 43 infants. Neither the effect of group (*F*_(1,41)_ = 2.35; *p* = 0.13, $\eta_{p}^{2}$ = 0.054) nor the familiarity x group interaction (*F*_(1,41)_ = 0.005; *p* = 0.94, $\eta_{p}^{2}$ < 0.001) were significant, failing to reveal an effect of prematurity.

To explore the potential effect of degree of prematurity, bilateral Pearson correlations were run between the familiarity effect (attesting segmentation) and infants’ gestational ages or birth-weights (unfortunately, detailed information about 1-min and 5-min Apgar scores and duration of hospital stay could not be obtained for this group of infants). When correcting for multiple comparisons (significant *p* at 0.025), the correlations between difference scores (OTs to target minus control words) and infants’ gestational age (r = −0.236; *p* = 0.33) and between difference scores and infants’ birth-weight (r = −0.480; *p* = 0.037) both failed to reach significance. Note that there is an unexpected tendency for infants with smaller birth-weights to perform better.

To explore the potential effect of degree of bilingualism, bilateral Pearson correlations were run between difference scores (OTs to target minus control words) and infants’ exposure to French. Correlations failed to reach significance when considering the full-term and preterm infants together (r = 0.092, *p* = 0.56), the full-terms alone (r = 0.177, *p* = 0.41), or the preterms alone (r = 0.005, *p* = 0.98).

Lastly, because the present monosyllabic word segmentation experiment has been run in four different populations (monolingual full-terms, [38]; monolingual preterms, [44]; bilingual full-terms and preterms, present study), we ran an additional repeated-measures ANOVA with mean OTs as the dependent measure, maturation (full-term versus preterm) and linguistic status (monolinguals versus bilinguals) as between-group factors, and familiarity (target versus control) as a within-group factor was conducted. The effect of familiarity was significant (*F*_(1,83)_ = 26.07; *p* < 0.001, $\eta_{p}^{2}$ = 0.239), infants having longer OTs to target (*M* = 9.03 s, *SD* = 3.16) than to control (*M* = 8.05 s, *SD* = 3.42) words, indicating successful word segmentation. There was a significant effect of maturation, with preterm infants (*M* = 9.49 s, *SD* = 3.55) having longer OTs than full-term infants (*M* = 7.61 s, *SD* = 2.79), suggesting differences in attentional abilities. All other effects (linguistic status: *F*_(1,83)_ = 0.269; *p* = 0.065, $\eta_{p}^{2}$ = 0.003; maturation x linguistic status: *F*_(1,83)_ = 0.491; *p* = 0.485, $\eta_{p}^{2}$ = 0.006) and interactions (familiarity x maturation: *F*_(1,83)_ = 0.139; *p* = 0.71, $\eta_{p}^{2}$ = 0.002; familiarity x linguistic status: *F*_(1,83)_ = 3.485; *p* = 0.065, $\eta_{p}^{2}$ = 0.040; familiarity x maturation x linguistic status: *F*_(1,83)_ = 0.076; *p* = 0.784, $\eta_{p}^{2}$ = 0.001) failed to reach significance.

## 4. Discussion

The goal of the present experiment was to explore whether early segmentation abilities are present in bilingual preterm and full-term 6-month-olds who have different linguistic environments but are exposed to French between 50 and 75% of the time on a daily basis and whether prematurity has an impact on their segmentation performance. They were tested on monosyllabic word segmentation in French, in the same experiment in which monolingual preterm 6-month-olds [44] and monolingual full-term 4- and 6-month-olds [38,44] were found to segment. The results show that, taken together, these bilingual preterm and full-term infants segment monosyllabic words from fluent speech. Analyses testing whether this segmentation ability was modulated by prematurity or language dominance failed to find significant effects. Only a weak and negative correlation with birth weight was found, which however appeared to be driven by an outlier preterm. In the following, we discuss these findings in more detail, starting with what it means for bilingual infants and then for prematurity.

With respect to bilingualism, our findings are in line with previous results having found that, by 6 to 8 months of age, full-term bilingual infants can segment words in their dominant [46,47,48,49] and non-dominant [46,48,49] languages, at the same age as their monolingual peers. They extend these results to a different group of infants, who share French as a language but vary considerably in their other language (and the linguistic distance between their two languages). This demonstrates bilingual segmentation abilities beyond the limited number of language pairs previously studied (English/Mandarin, English–Spanish, French/English, and Spanish/Catalan), and to a more mixed language environment than the one in which French/English bilinguals in the Montreal area or Spanish/Catalan in the Barcelona area are likely encountering. Moreover, since the present study used the exact same task as the one used to test monolingual full-term and preterm infants [38,44], we ran a joint analysis to make a direct comparison between monolingual and bilingual infants. It revealed a non-significant interaction between segmentation performance and language status, thus failing to find a difference in performance between monolinguals and bilinguals. This supports the previous finding of segmentation abilities found at the same age in monolingual and bilingual infants. Lastly, we failed to find effects of language dominance on segmentation performance, in line with the previous studies having explored this issue [46,49]. However, our examination of the potential effect of language dominance is limited by the fact that we did not test French-non-dominant bilinguals, and thus that our range only spanned from 50 to 75% of French input.

Why did our balanced or French-dominant bilinguals succeed as well as monolinguals in our monosyllabic words segmentation task? One possibility is that the amount of input in French they have received by 6 months is enough to elicit acquisition of basic segmentation skills. Indirect evidence for this idea comes from the finding that 4-month-old monolingual infants, who have also had less experience with French than 6-month-old monolinguals, show successful segmentation of the same monosyllabic words [44]. This is also consistent with previous evidence of French–German bilingual infants showing a trochaic bias at 6 months like monolingual German-learning 6-month-olds, although they received less input in German [53]. A second possibility is that the words to-be-segmented, being monosyllabic words, were easy to segment, as supported by independent evidence suggesting that the syllabic unit is a default processing unit. Indeed, syllables are better processed than smaller (moraic units) or larger (multisyllabic feet) units, both in newborn infants not yet tuned to the processing of a specific language [54,55] and in adults across three rhythmically different languages, namely English, French, and Japanese [56]. Moreover, across languages, segmentation of monosyllabic words is always found earlier or at the same age as segmentation of larger or smaller units [36,37,38,57]. This status of default processing unit might contribute to the success of the bilingual infants in our study, at their performance at a similar level as their monolingual peers. Future studies will be needed to assess whether bilingualism might pose a bigger challenge for the segmentation of more complex word structures.

With respect to prematurity, our results do not show an effect of prematurity status, indicating no differences in segmentation abilities between bilingual preterm and full-term infants. Within the preterms, there was no effect of gestational age, suggesting that even the more premature of the preterms (who also constitute the majority of the group) were segmenting the words. Lastly, in the joint analysis including the data from monolinguals [38,44], we found a significant impact of prematurity status on orientation times, with longer times for the preterms (although that effect did not impact the segmentation effect). This effect might signal differences in preterm infants’ attentional abilities, in their motor abilities, or in their processing of audio–visual displays. It is in line with a recent finding showing that preterm infants have shorter looking times than full-terms in an audiovisual experiment at 8 months of postnatal age [58]. Lastly, our results on monosyllabic word segmentation show no interaction between prematurity and bilingualism; none of these factors having a significantly detrimental effect on performance. Hence, bilingualism did not pose an additional difficulty for our preterms. This finding is in line with recent evidence that bilingualism is not a problem for lexical acquisition (see review by [45]), though it is to our knowledge the first time that this is established for preterm infants.

## 5. Conclusions

In conclusion, the present study shows segmentation abilities at 6 months of postnatal age in bilingual preterm and full-term infants, with no significant performance difference compared to equivalent monolingual preterm and full-term groups. It is the first study showing such effects for bilingual preterms and, as such, shows that bilingual parents of preterm infants might not have to fear to raise their children bilingually. However, our findings have some limitations in at least two ways. First, they are found for the segmentation of monosyllabic words, which in French are known to be segmented easily and at an early age. Therefore, they may not extend to the segmentation of more complex word structures. Second, our findings regarding prematurity pertain to preterm infants in good health. Therefore, they may not extend to preterm infants with severe neonatal morbidities. These issues should be evaluated in future studies, which will also have to test preterms on other language-related abilities and at different stages of their linguistic development. Moreover, it will be important to examine potential performance differences in bilingual infants and preterm infants in association with brain development and organization. For example, researchers could employ ERP protocols similar to those used in prior studies on early word segmentation by monolingual full-term infants, which revealed individual differences in ERP response polarity that correlated with vocabulary acquisition at a later stage [41,42,59,60].

## Figures and Tables

**Table 1:** Neonatal characteristics of preterm infants.

Neonatal History (Values Correspond to Mean, SD, and Range)	Included (N = 19)	Excluded (N = 9)	Difference (1-Tailed *t*-Tests)
Gestational age (wk)	30.1 (3.1) range: 26–35	32.17 (3.7) range: 25–35	*n.s.*
EPT (<28 wks, n)	7	2	
VPT (28 < 32)	6	1	
MPT (32 < 34)	2	1	
LPT (34 < 37)	4	5	
Birth weight (g)	1.359 (454) range: 670–2420	1.605 (603) range: 780–2300	*n.s.*

*n.s*.: Non-significant.

**Table 2:** Language background of preterm and full-term participants.

	Preterm		Full-Term	
	(N = 19)		(N = 24)	
Language	Balanced	French-Dominant	Balanced	French-Dominant
	(*n*)	(*n*)	(*n*)	(*n*)
Arabic	4	5	1	3
Azerbaijani				1
Bambara	1			
Bulgarian			1	
Creole (French Guyana)		2		
Italian	1	1		3
Japanese				1
Kasshonke (Mali)				1
Korean				1
Koyaka (Ivory Coast)				1
Loma (Guinea)		1		
Malagasy (Madagascar)	1			
Mandarin Chinese	1		1	
Persian				1
Polish				1
Portuguese			1	1
Romanian				2
Russian	1			2
Slovak				1
Soninke				1
Spanish	1			

## Data Availability

Data will be made available upon reasonable request.
